# Natural CO_2_ seeps reveal adaptive potential to ocean acidification in fish

**DOI:** 10.1111/eva.13239

**Published:** 2021-05-05

**Authors:** Natalia Petit‐Marty, Ivan Nagelkerken, Sean D. Connell, Celia Schunter

**Affiliations:** ^1^ Swire Institute of Marine Science School of Biological Sciences The University of Hong Kong Hong Kong Hong Kong SAR; ^2^ Southern Seas Ecology Laboratories School of Biological Sciences and the Environment Institute DX 650 418 The University of Adelaide Adelaide SA Australia

**Keywords:** adaptation, balancing selection, gene expression, global change, standing genetic variation, transcriptomics

## Abstract

Volcanic CO_2_ seeps are natural laboratories that can provide insights into the adaptation of species to ocean acidification. While many species are challenged by reduced‐pH levels, some species benefit from the altered environment and thrive. Here, we explore the molecular mechanisms of adaptation to ocean acidification in a population of a temperate fish species that experiences increased population sizes under elevated CO_2_. Fish from CO_2_ seeps exhibited an overall increased gene expression in gonad tissue compared with those from ambient CO_2_ sites. Up‐regulated genes at CO_2_ seeps are possible targets of adaptive selection as they can directly influence the physiological performance of fishes exposed to ocean acidification. Most of the up‐regulated genes at seeps were functionally involved in the maintenance of pH homeostasis and increased metabolism, and presented a deviation from neutral evolution expectations in their patterns of DNA polymorphisms, providing evidence for adaptive selection to ocean acidification. The targets of this adaptive selection are likely regulatory sequences responsible for the increased expression of these genes, which would allow a fine‐tuned physiological regulation to maintain homeostasis and thrive at CO_2_ seeps. Our findings reveal that standing genetic variation in DNA sequences regulating the expression of genes in response to a reduced‐pH environment could provide for adaptive potential to near‐future ocean acidification in fishes. Moreover, with this study we provide a forthright methodology combining transcriptomics and genomics, which can be applied to infer the adaptive potential to different environmental conditions in wild marine populations.

## 1 INTRODUCTION

Human‐driven global change is challenging the scientific community to understand how marine species might adapt to predicted environmental conditions in the near future (e.g., hypoxia, ocean warming, and ocean acidification). The effects of the uptake of anthropogenic atmospheric CO_2_ by oceans propagate across the biological hierarchy, from changes in the building blocks of life at nanoscales (Leung et al., 2020) to organismal physiology and behavior (Strader et al., 2020) through to ecosystem processes and their properties (Nagelkerken et al., 2016; Sunday et al., 2017; Teixidó et al., 2018). These higher‐order changes alter biodiversity, species abundances and composition, and how energy propagates through food webs (Doubleday et al., 2019; Martínez‐Crego et al., 2020; Nagelkerken et al., 2020). Some observations of wild populations are surprising because they reveal species thriving rather than declining in naturally acidified environments (Connell et al., 2017; Nagelkerken et al., 2017), but we still have little understanding of how adjustments to climate change at organismal level are linked to adaptive processes at the population level.

To survive in a reduced‐pH environment, marine organisms have to adjust their physiology, which, at the molecular level, is achieved by modifying the expression of genes. At the organismal level, we are learning that reduced‐pH environments alter the expression of many genes (hundreds or more, e.g., Evans et al., 2017; Schunter et al., 2016) mostly affecting traits related to calcification, metabolism, acid–base regulation, stress responses, and behavior (Strader et al., 2020). Such changes in gene expression can reflect the capacity of species to respond to environmental variability (i.e., physiological acclimatization; Schlichting & Smith, 2002) but could also indicate responses with negative effects on the fitness of individuals, which would compromise the long‐term survival of the species (Kelly & Hofmann, 2013; Strader et al., 2020). At the population level, little is known about the potential of the wild species to long‐term adaptation to ocean acidification, given that naturally pH‐reduced coastal marine environments are not common (Kelly & Hofmann, 2013; Palumbi et al., 2019). This knowledge from natural environments is critical for the understanding of the adaptive potential to environmental stressors in wild populations because the inherent buffering processes within naturally complex systems (Connell & Ghedini, 2015) reduce the applicability of observations from laboratory experiments to natural ecosystems (Goldenberg et al., 2018).

Long‐term adaptation happens when genetic changes alter phenotypes improving the fitness of the individuals within the environment they live in. Alteration to individual fitness is driven by environmental or ecological changes that exert selective pressures on populations. The fitness differential among individuals of a population fuels natural selection allowing long‐term adaptation to occur (Orr, 2009). If a genetic change improves the fitness of an individual (beneficial or adaptive mutation), natural selection will increase the frequency of this change toward reaching the whole population (i.e., complete or local adaptation, Orr, 2009). However, the fitness effect of a mutation may vary spatially among different environments inhabited by a single population with random mating. When this happens natural selection is therefore spatially heterogeneous (Hedrick, 2006, 2007; Levene, 1953), implying that within one generation, a mutation could be positively selected if the carriers reside in one area (i.e., individuals with the mutation will contribute more to the next generation) but selected against or not selected if they reside in another (i.e., individuals with the mutation will contribute less to the next generation). This process is especially important in marine species as most of them distribute across heterogeneous environments and have highly dispersive larvae (Bernatchez, 2016; Kelly & Hofmann, 2013; Palumbi et al., 2019).

Spatially heterogeneous selection was first proposed by Levene (1953) to explain how genetic variation can be maintained within a population distributed across different ecological niches. Currently, this kind of selection is commonly known as a form of balancing selection (Charlesworth, 2006; Hedrick, 2006, 2007). Balancing selection refers to different selective processes that result in the maintenance of genetic diversity within populations (Charlesworth, 2006; Fijarczyk & Babik, 2015; Hedrick, 2007). Hence, the importance of balancing selection as a mechanism of preserving the adaptive potential of marine species to climate change has been highlighted in recent studies (Bernatchez, 2016; Bitter et al., 2019; Brennan et al., 2019; Kelly & Hofmann, 2013; Palumbi et al., 2019). Balancing selection can be identified as it leaves a footprint in the pattern of neutral nucleotide polymorphisms in the nearby genomic regions of mutations under selection, mostly characterized by an excess of intermediate‐frequency alleles (Charlesworth, 2006; Fijarczyk & Babik, 2015; Hedrick, 2007). Evidence of balancing selection's capacity to maintain adaptive mutations to ocean acidification has been demonstrated in the sea urchins (*Strongylocentrotus purpuratus*; Brennan et al., 2019) and mussels (*Mytilus galloprovincialis*; Bitter et al., 2019), but not yet for fishes.

Understanding how fitness is connected to genotypes is a ‘holy grail’ toward enabling predictions on the adaptive potential of species to environmental change in general and climate change in particular. Changes in gene expression due to a change in the environment can provide insights into the fitness benefits of a genotype because the responding genes are likely targets of natural selection (Kelly & Hofmann, 2013; Schlichting & Smith, 2002). The transcriptome of individuals in a given environmental condition could be seen as a first representation of their phenotypes. Genes with increased transcription under environmental change are especially relevant as they are likely triggering regulatory circuits enhancing or suppressing the transcription of other genes (Yosef & Regev, 2011). Hence, if an environmental change persists in time, genetic changes in those responding genes can allow faster or improved responses, which are then expected to be positively selected and increase their frequencies in the population. Therefore, transcriptomics has become a powerful tool to investigate changes in expression of genes underlying the responses to environmental stressors and can aid in revealing the adaptive mechanisms of these responses.

Volcanic CO_2_ seeps provide a unique opportunity to study the long‐term adaptation to ocean acidification of marine species. CO_2_ seeps exhibit a net resource enrichment that benefits the whole trophic chain (Doubleday et al., 2019; Martínez‐Crego et al., 2020; Nagelkerken et al., 2017) and can boost population densities of various fish species (Ferreira et al., 2018; Nagelkerken et al., 2016). While the net resource enrichment at CO_2_ seeps can be a positive selective pressure for adaptation to reduced pH by marine species, the molecular mechanisms underlying the adaptation to ocean acidification remain elusive and are still unknown in wild fish populations. Here, we use transcriptomics to discover candidate genes of adaptive selection in a naturally reduced‐pH environment to infer potential mechanisms of adaptation to ocean acidification in a fish species. We study a fish (Common triplefin, *Forsterygion lapillum* Hardy, 1989) that experiences doubling in its population size on temperate rocky reefs at natural CO_2_ vents at White Island, New Zealand, as a model species whose populations benefit from ocean acidification.

## 2 METHODS

### 2.1 Sampling

Two CO_2_ seeps at White Island (New Zealand) are located within temperate rocky reef habitats at 6 to 8 m of depth and have an average pH reduction of about −0.17 and −0.24 units, respectively, across years compared with ambient pH seawater (Nagelkerken et al., 2021), reflective of representative concentration pathway emission scenarios 4.5–6.0 (Bopp et al., 2013). Twenty individuals of the Common triplefin *Forsterygion lapillum* were collected on SCUBA with hand nets from rocky reefs habitats at the volcanic vents and adjacent control sites of White Island (New Zealand) in January 2019. Ten individuals were caught at the underwater CO_2_ seeps with reduced pH (hereafter called ‘seeps’) and ten in neighboring zones (~25 m away) with ambient pH (hereafter called ‘control’). Individuals were measured for total body length. Their gonad tissue was dissected out immediately and placed in RNAlater (Invitrogen Inc.) and eventually frozen at −80 C for further processing.

### 2.2 RNA sequencing and processing

RNA extractions were performed on each gonad tissue using a Qiagen RNeasy Micro Kit according to the manufacturer's instructions including DNAse I treatment. Quality checks were performed on a Qubit and an Agilent Bioanalyzer 2100, and all RNA had a RIN above 8. RNA‐seq libraries were built for the twenty Common triplefin individuals with Illumina TruSeq kits and randomized in barcoding to be sequenced at 150 bp paired‐end on an Illumina NovaSeq at the Center of Panoramic Sciences at the University of Hong Kong. After sequencing, the average number of paired‐end RNA‐seq reads per individual was 39.1 ± 2 M. Additionally, one RNA‐seq library from gonads of a wild‐type zebrafish (*Danio rerio*) was downloaded from the NCBI SRA database (Accession no. SRX4149436; *N*_reads_ = 16.8 M) and was used as a control for the de novo assembly and annotation processes.

All sequenced reads were trimmed using the program Trimmomatic (Bolger et al., 2014) eliminating sequencing adaptors and low‐quality positions (sliding window 4:15). Unpaired reads and reads with a final length lower than 40 nts were removed from further analysis. The read quality was assessed using FastQC (Andrews, 2010) and summarized with MultiQC (Ewels et al., 2016) with the average Phred score of 36 after trimming. The final average number of Common triplefin reads per sample was 22.7 ± 1.3 M.

### 2.3 Sexing of the individuals

Given the small size of the Common triplefin gonads, the sex of the fish was determined by using the levels of expression of zona pellucida‐related genes (zp genes). We looked for highly expressed zp genes in female gonads of zebrafish (*Danio* *rerio*) in the Bgee DataBase (https://bgee.org/). Then, we retrieved the protein sequences of the orthologous genes of these zp genes for four phylogenetically closest species to the triplefin with sequenced genomes (from Eupercaria group of species in Ensembl genome phylogeny: *Labrus bergylta* (ballan wrasse), *Cottoperca gobio* (channel bull blenny), *Gasterosteus aculeatus* (stickleback), and *Larimichthys crocea* (large yellow croaker) from Ensembl orthologous gene database (https://www.ensembl.org). Then, RNA‐seq libraries of the Common triplefins were transformed to FASTA format using seqKit (Shen et al., 2016) and blasted against a database with the orthologous zp genes, using BLASTn software with the task dc‐megablast (Smith & Waterman, 1981). The number of reads with significant blast hits for each sample was taken as first evidence to separate males from females. An RNA‐seq library presenting a high number of blast hits (putative female) was assembled and annotated. Then, the levels of gene expression of the orthologous zp genes were quantified for all samples following the methodology described below. We found striking concordance between the number of blast hits and the number of quantified reads for zp genes and classified the samples according to these results (Table S1). The 20 fish randomly collected represented ten females (six from control sites and four from CO_2_ seeps) and ten males (four from control sites and six from CO_2_ seeps).

### 2.4 Primary transcriptome assembly

De novo transcriptome assemblies were performed using the software Trinity v2 with default options (Grabherr et al., 2011) separately for (1) one male and one female from control, (2) one male and one female from seeps, and (3) one sample of zebrafish gonads (assembly control). Assembled transcripts were translated into proteins and coding nucleotide sequences using the software Transdecoder v5 (Haas & Papanicolaou, 2015). The primary transcriptome assembly steps are shown in the first part of Figure S1 and basic statistics in Table S2.

### 2.5 Primary assembly postprocessing and annotation

The longest protein sequences of the assembled transcripts for the four triplefin samples (min. 100 aa) were blasted against the Ensembl Release 99 protein databases (https://www.ensembl.org) of the four phylogenetically closest species with sequenced genomes: *Labrus bergylta*, *Cottoperca gobio*, *Gasterosteus aculeatus*, and *Larimichthys crocea*. The same was performed for the control sample of zebrafish, but the primary assembly was blasted against the zebrafish Ensembl protein database.

To eliminate transcripts redundancy produced by alternative transcripts and orthologous genes, we filtered the BLASTp results following these steps (second part of Figure S1):

1. Each Common triplefin assembly (*N* = 4) was blasted (BLASTp) separately against each reference protein database (*N* = 4) with a cutoff of e‐value <10^−6^ and a percentage of sequence identity >70%.
2. For each BLASTp result to a reference species, we merged the results of the four samples grouping them by the ID of the reference protein and keeping the ID of the assembled transcript whose blast alignment covered the highest length of a reference protein. The number of assembled transcripts is presented in Table S3 at protein/transcript level by each assembly and at the gene level for the merged assembly in Table S4. The comparison with zebrafish showed that the postprocessing method using blast analyses against phylogenetically related, though distant, species, results in nearly the same number of assembled transcripts as expected in a gonad sample from zebrafish (Table S3).
3. The BLASTp results for each reference species (*N* = 4) were merged grouping the results by a unique gene ID represented by the orthologous gene in stickleback (the best‐annotated genome of the four reference species), and keeping the ID of the assembled transcript whose blast alignment covered the highest length of a reference protein sequence. Merging different assemblies and using different reference genomes for annotation give a higher number and quality of assembled coding sequences than using single samples and only one reference species (Tables S3 and S4).

The reference transcriptome assembly has 13,630 nonredundant genes with protein sequences that can be aligned to reference sequences in more than 50% of their length (82% covering more than 90% and 64% covering the full length; Table S5). Seventy‐eight percent of the genes in this reference assembly has annotated orthologous genes in the four related species, while only 4% have annotated orthologous genes in one single species. Thus, the Common triplefin reference transcriptome presents great reliability in terms of functional annotation, with two parameters useful to assess their confidence: the number of related species with significant protein homology, and the length of the recovered transcript (Table S5).

### 2.6 Gene expression quantification and differential gene expression analysis

The quantification of expression of the genes was performed using Salmon v1.2 (Patro et al., 2017). To obtain TPM (transcripts per million) estimates, we used Salmon's options ‐‐validatMapping ‐‐GC, and bootstrapping (‐‐numBootstraps 10,000). DESeq2 v2 (Love et al., 2014) was used to test for differential gene expression between control and seep individuals. Normalized read counts were calculated by sample and gene from Salmon expression quantification results. A likelihood‐ratio test (LRT) was used to test for significant differential gene expression contrasting a model including environmental pH factor (pH) plus the covariants (sex and body size) and the interaction between pH and the covariants, against an alternative model without the environmental pH factor. *p*‐values from the differential expression analysis were adjusted for multiple testing with the Benjamini–Hochberg procedure (Love et al., 2014). To obtain a set of genes to be used as a neutral reference in the selection analysis, we searched for genes without evidence of differential expression. This was done by means of Wald tests contrasting the expression of genes among sexes (*N* = 8916 DE genes, nonadjusted *p*‐value < 0.05) and among pH environments without taking into account the variance in sex and body length (*N* = 8248 DE genes, nonadjusted *p*‐value < 0.05). We used an ANCOVA test to investigate the effect of pH on the overall level of gene expression controlling for the variation in body size and sex. These gene expression levels represent the average of normalized read counts across all genes, as computed by DESeq2. Two‐way ANOVAs (with fixed factors pH and sex) were used to test differences in levels of gene expression for the whole dataset and by functional keywords categories (homeostasis, energy production, protein production, protein binding, actin, and signal transduction). Statistical analyses were performed in R (R Core Team, 2020).

### 2.7 Functional annotation and enrichment analysis of up‐regulated genes

Gene Ontology terms (GO) were obtained for the orthologous genes in stickleback genome from Ensembl Release 99 Database (https://www.ensembl.org). The functional enrichment analysis was performed by counting the number of genes for each GO category in the up‐regulated gene dataset and comparing this with the number of annotated genes in each category in the stickleback genome. Tests for differences used Fisher's exact test where *p*‐values were adjusted via Bonferroni correction. Functional annotation for genes without GO annotation in stickleback Ensembl database (*N* = 8) was obtained from orthologous protein annotation in UniProt for stickleback or zebrafish. Functional keywords were extracted from the GO terms of each gene (Table S6). Information on genes related to X‐linked disease in humans was retrieved from the GeneCards webpage (https://www.genecards.org).

### 2.8 Natural selection analysis

Patterns of nucleotide variation across genome regions are informative regarding the presence of natural selection. The folded site frequency spectrum (fSFS) shows the frequency of the minor allele (MAF) in a dataset and is used to find deviations of the pattern of nucleotide diversity related to that expected under a neutral model. Thus, we evaluated the impact of natural selection on genes expressed at significantly higher levels in individuals from CO_2_ seeps (up‐regulated genes) by comparing the fSFS of these genes with a set of neutral genes. We considered neutral genes the set of genes (*N* = 106) without evidence of different levels of expression between seeps and control, or between males and females (Wald test *p*‐value > 0.05), and not functionally related to up‐regulated genes (i.e., not containing any of the functional keywords listed in Table S6). As we are searching for genetic variation in differentially expressed genes and the methods for calling single nucleotide polymorphisms (SNPs) are based on read coverage, we only call SNPs for genes with evidence of being expressed in all individuals. For this, we mapped all filtered RNA‐seq reads against the datasets of up‐regulated and neutral reference coding sequences using MagicBlast v1.5 with default parameters (Boratyn et al., 2019). The obtained bam files were then used to guide the assemblies of up‐regulated and neutral genes for each sample with Trinity v2 using the option –genome_guided, and a minimum number of read coverage of 3 (Grabherr et al., 2011). We commonly assembled transcripts in all samples for 39 up‐regulated and 97 neutral genes. To perform the SNP calling, we piled up the bam files using SAMTools (Li et al., 2009) and then used VarScan mpileup software (Koboldt et al., 2009) with the parameters: ‐‐min‐coverage = 10, ‐‐min‐reads 2 = 3, ‐‐min‐avg‐qual = 15, ‐‐min‐var‐freq = 0.05, ‐‐min‐freq‐for‐hom = 0.75, ‐‐*p*‐value = 0.99. Alternatively, we called SNPs by aligning the longest assembled transcripts obtained for each gene and sample using MUSCLE (Edgar, 2004) obtaining the variant positions with a custom perl script that counts the number of the different nucleotides (A, C, T, and G) in each position of the alignment. This alternative method (aligning method) lacks power to detect low‐frequency SNPs. However, aligning complete assembled transcripts minimizes the probability of false SNP discovery, and therefore, we use this method for the verification of the detected high‐frequency SNPs with VarScan.

We identified 265 and 1116 SNPs in up‐regulated and neutral genes, respectively, with VarScan. We did not find a decrease in the average number of low‐frequency SNPs (MAF < 10%) in control (low expression) when compared to seeps (high‐expression) in candidate genes (Table S7), indicating that the SNP calling is not affected by the differences in expression. Basic statistics of the discovered SNPs by the two methods are presented in Table S7. To get the fSFS, we only took biallelic SNPs with allele information in all twenty fish into account. fSFS was calculated for up‐regulated genes, neutral genes, and the up‐regulated genes grouped by their functional category. All coding SNPs with intermediate frequency were also detected by the aligning method of SNP detection, and classified in synonymous or nonsynonymous by aligning the assembled transcripts from the twenty fish to the coding sequence of the orthologous species with high similarity (see Primary assembly postprocessing and annotation). Differences among fSFSs were tested using one‐sided two‐sample Kolmogorov–Smirnov tests as implemented in R (R Core Team, 2020). Differences in SNP allele frequency between seeps and control were tested using Fisher's exact test, and the *q*‐value R package (R Core Team, 2020) was used to correct for multiple testing.

## 3 RESULTS

### 3.1 Overall elevated gene expression in fish from natural CO_2_ seeps

High variability in gene expression is expected from wild populations, and therefore, we first analyzed the effect of elevated CO_2_ on the overall gene expression levels, taking into account individual traits that can influence this. The average level of gene expression was significantly higher in fish from seeps than from control (analysis of covariance, *p* = 0.016), and this covaried negatively with fish body length (*p* = 0.006; Table S8; Figure S2). Sex did not have a significant effect on overall gene expression (Table S8, *p* > 0.05), although the highest levels of expression were observed in males from seeps (Figure S2B).

To identify all genes with significantly elevated expression in fish from CO_2_ seeps versus those from controls, a LRT was performed. For each gene, we calculated the likelihood of the differences in gene expression in a model including the three factors (sex, body length, and environmental pH), and in a model not including the pH as a factor. For 107 genes, the model including environmental pH better explained the differential expression than the alternative model excluding this factor (LRT, adjusted *p* < 0.05; Table S9). Sixty‐six of these genes exhibit higher levels of expression (up‐regulated) in seep individuals (Table 1). Up‐regulated genes are potential candidates as targets of adaptive selection to ocean acidification and further analyses focused on these.

**TABLE 1:** List of the 66 *up*‐*regulated* genes found in Common triplefin living at CO_2_ seeps with information of the confidence of the assembled transcripts (*N* species: number of related species where high similarity proteins were found; % recovered protein: percentage of the protein sequence that was aligned with the orthologous reference protein), and its functions as described by functional keywords

				Main functional keywords					
Orthologous gene ID	Gene name	*N* species	% Recovered protein	Homeostasis	Energy production	Protein production	Actin	Signal transduction	Protein binding
ENSGACG00000000075	BICD2	4	100				X		
ENSGACG00000000342	zgc:56235	4	100	X					
ENSGACG00000000451	NR4A1	4	100	X		X			
ENSGACG00000001180	n/d	4	100	X	X				
ENSGACG00000001528	n/d	3	61		X		X		X
ENSGACG00000001557	cyb561d2	4	100	X					
ENSGACG00000001638	cyp2ad2‐207	3	100	X					
ENSGACG00000001993	MACF1	3	73	X			X		X
ENSGACG00000002095	fbxo45	4	99						X
ENSGACG00000002441	bfsp2	2	100				X		
ENSGACG00000002699	n/d	1	99			X			
ENSGACG00000003060	n/d	3	100						X
ENSGACG00000003199	emx2	4	100			X			
ENSGACG00000003382	RASSF10	4	100					X	
ENSGACG00000003468	ankrd10b	4	100						X
ENSGACG00000003738	chst2b	4	100		X				
ENSGACG00000004230	RPL5	4	100			X			
ENSGACG00000004583	fsta	4	100						X
ENSGACG00000004699	rpl35a	3	99	X		X			
ENSGACG00000005133	n/d	3	100	X					
ENSGACG00000005383	rbm24a	3	100	X		X			
ENSGACG00000005442	fads2	4	100		X				
ENSGACG00000005784	aoc2	4	100	X					
ENSGACG00000006523	RPL27A	4	99			X			
ENSGACG00000006809	tdg.1	4	100						
ENSGACG00000006865	lrp2b	3	54	X					X
ENSGACG00000007184	EMP1	4	100	X					
ENSGACG00000008035	mmp9	4	100	X					
ENSGACG00000008464	ank1a	4	78					X	X
ENSGACG00000008643	b3gnt3.4	3	99	X	X				
ENSGACG00000009136	trpv4	4	100	X					
ENSGACG00000009372	RARG	2	100	X		X			
ENSGACG00000009865	rhag	4	100	X					
ENSGACG00000010000	n/d	4	65		X		X		
ENSGACG00000010010	EZR	4	100				X		
ENSGACG00000010732	n/d	1	100	X					
ENSGACG00000010742	fam174b	3	100	X					
ENSGACG00000010749	slco3a1	4	100	X					
ENSGACG00000011061	agmat	3	93	X					
ENSGACG00000011391	dnmt3ab	4	100		X	X			
ENSGACG00000012183	chp1	4	100	X					
ENSGACG00000012484	zgc:171772	4	100			X			
ENSGACG00000013112	rgl2	4	100					X	
ENSGACG00000013516	tmod4	4	100				X		
ENSGACG00000013530	hsp90ab1	4	100	X	X				X
ENSGACG00000013597	si:ch211‐107n13.1	4	100				X		
ENSGACG00000013647	atp1b1a	3	100	X	X				
ENSGACG00000014242	dpy19 l3	3	87	X					
ENSGACG00000014358	FILIP1L	4	99	X					
ENSGACG00000014362	n/d	1	100		X				
ENSGACG00000014465	klf17	1	100	X		X			
ENSGACG00000014559	rpl3	4	100			X			
ENSGACG00000015302	rasa3	4	100	X				X	
ENSGACG00000015691	adma	4	100					X	
ENSGACG00000017200	taldo1	4	99		X				
ENSGACG00000017423	hypk	4	99				X		
ENSGACG00000017730	VANGL2	4	100	X					
ENSGACG00000018488	n/d	4	100	X					
ENSGACG00000018525	n/d	4	95	X		X			
ENSGACG00000019020	celsr1a	4	100	X				X	
ENSGACG00000019118	faah2b	2	100		X				
ENSGACG00000019640	si:zfos‐2326c3.2	4	76		X				
ENSGACG00000019847	cct7	4	100		X				X
ENSGACG00000019909	ccnd2b	4	100						
ENSGACG00000020331	ophn1	4	99					X	
ENSGACG00000020814	si:dkeyp‐23e4.3	3	54					X	

### 3.2 Functional analyses of candidate genes for adaptation to ocean acidification

**FIGURE 1:**
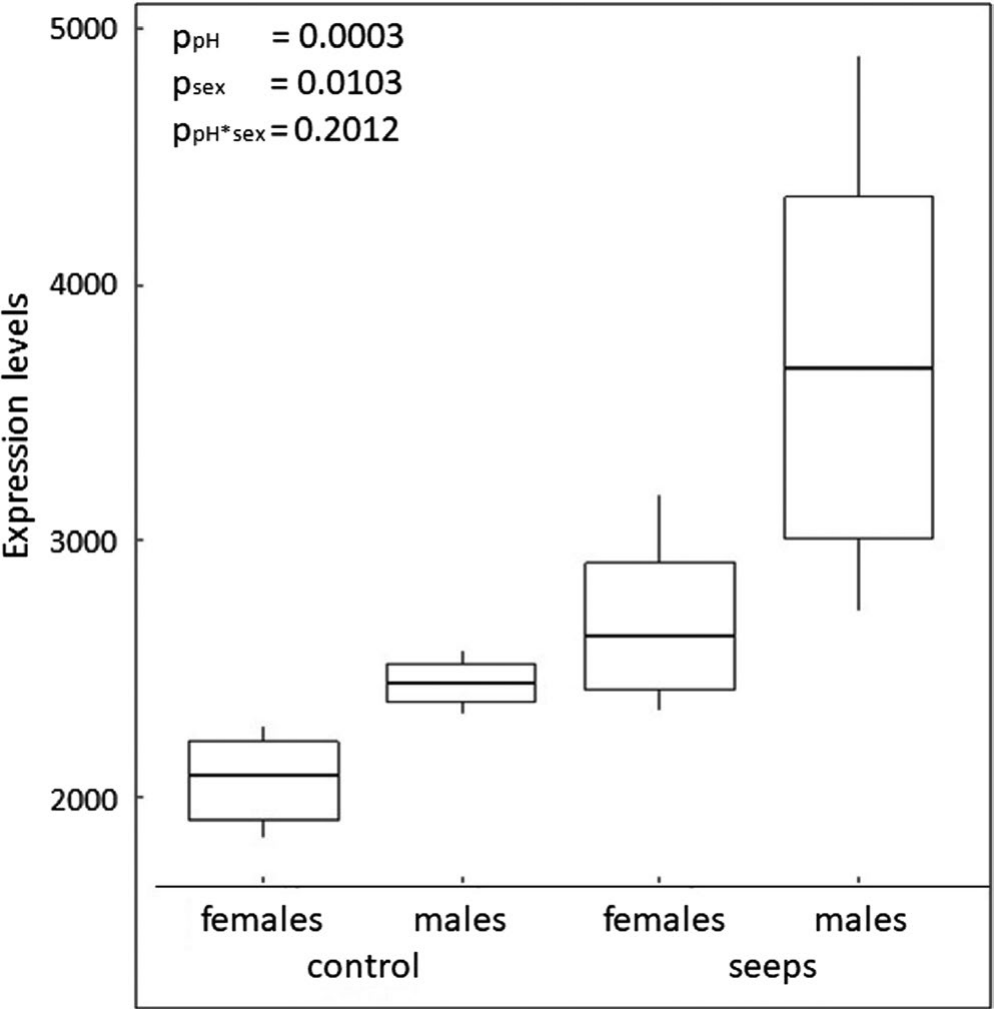
Boxplot of the differences in levels of gene expression in all up‐regulated genes of male and female fish from natural CO_2_ vents and their controls, expressed as normalized reads counts. Line: median, lower and upper hinges correspond to the 25th and 75th percentiles, and whiskers are 1.5 * IQR

The expression of the 66 up‐regulated genes in seeps was on average higher in males than in females (Figure 1). We found only three genes with clear evidence of being highly expressed in seep females, but less expressed in seep males (*EMP1*, *tmod4*, *hypk*; Figure S3A‐C), and one with similar elevated levels of expression in both sexes at seeps (*taldo1*; Figure S3D).

The functional annotation of these 66 genes included 260 different GO terms (Table S10), while the functional enrichment analysis showed four related GO terms significantly enriched for these genes: *intracellular* and *ribosome* (Cellular Component), *translation* (Biological Process), and *structural component of ribosome* (Molecular Function) (adjusted *p*‐values = 0.030, 0.027, 0.028, and 0.005, respectively). There was no evidence of enrichment in genes related to gonad‐specific functions, but we found five genes related to hormonal activity (Table S10). Moreover, two genes are associated with X‐linked diseases in humans, *EMP1* and *ophn1*. *EMP1* is one of the three genes showing average elevated values of gene expression in females from seeps but also presented a high variance among individuals (Figure S3C). The opposite was observed for *ophn1* gene, with highest expression in males from seeps and less variance among individuals (Figure S3E).

Sixty‐four out of the 66 genes could be annotated and grouped by common functional keywords (FK; Table S6) and can be represented by eleven FK that we further grouped into six general main functions (Table 1): (1) intra‐ and extracellular maintenance of homeostasis (hereafter homeostasis; FK: membrane, ion binding, and blood; *N* = 32); (2) energy production (FK: metabolic, ATP, transferase; *N* = 14); (3) protein production (FK: regulation of gene expression and translation; *N* = 13); (4) FK: protein binding (*N* = 10); (5) FK: actin (*N* = 9); and (6) FK: signal transduction (*N* = 8). Overall, males from seeps displayed the highest values of gene expression for all six main functions and the gene expression was significantly different among sexes in four main functions (protein production, energy production, signal transduction, and protein binding; Figure S4A‐F). One clear example of this sex‐biased gene expression is the gene *ophn1* (Figure S3E) for which there is also evidence of sex‐related functions in zebrafish. This gene is functionally related to the GO term signal transduction, which indicates that it produces changes in the activity or state of a cell when an external or internal stimulus is received. Thus, the elevated expression of *ophn1* could enable the differential elevated gene expression in males from CO_2_ vents.

### 3.3 Natural selection footprint in candidate genes for adaptation to ocean acidification

**FIGURE 2:**
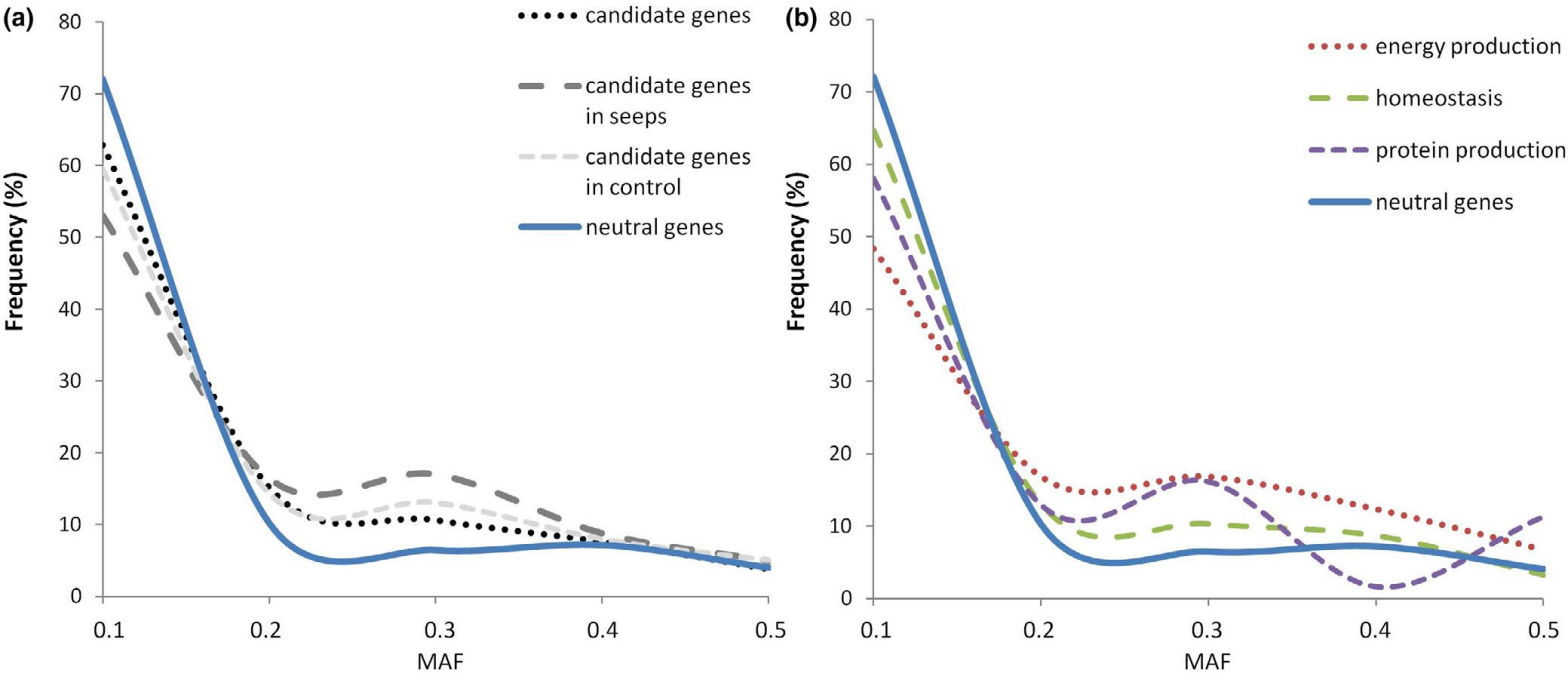
Folded site frequency spectrum (fSFS) of the minor allele frequency (MAF) for (a) candidate genes for adaptation to ocean acidification (all, seeps, and control fish) compared with neutral genes, and (b) candidate genes grouped by functional categories compared with neutral genes. The numbers of SNPs were: neutral = 1116, candidate (all) = 265, candidate (seeps) = 190, candidate (control) = 238, homeostasis = 184, energy production = 90, and protein production = 63

Fish larvae of the same age cohort recruit across control and adjacent seep sites at White Island, and hence, adaptive selection leading to local adaptation at CO_2_ seep environments is counteracted by the dispersal of larvae (i.e., balancing selection). We tested this hypothesis by comparing the fSFS of the candidate genes to adaptive selection (i.e., the up‐regulated genes in seeps) against the fSFS obtained from a set of neutral genes (i.e., without any evidence of differential expression in seeps). To minimize SNP miss‐calling (see Methods), we only obtained fSFS for genes in which full‐length transcripts could be assembled in all 20 samples (*N* = 39 and 97, for candidate and neutral genes, respectively). No significant difference was found between fSFSs from seep and control sites (one‐side K‐S test, *p* = 0.818; Figure 2a). However, a significant skew toward higher minor allele frequencies (MAFs) was found in the candidate when compared to the neutral genes for the whole population (seep + control; one‐side K‐S test, *p* = 0.041; Figure 2 A), as can be expected under a balancing selection scenario. In agreement with this balancing selection scenario, we found higher values of average heterozygosity in candidate compared with neutral genes (Table S7).

To investigate whether this skew toward higher MAF in the frequency spectrum could be related to some of the main functional categories of the candidate genes, we grouped the genes based on their functions [homeostasis (*N* = 20), energy production (*N* = 9), and protein production (*N* = 12)] and compared their fSFSs with that of neutral genes. These three functional categories showed significant deviations in comparison with the expected neutral fSFS (one‐side K‐S test, *p* = 0.007 for energy and protein, and *p* = 0.041 for homeostasis), with the excess of intermediate‐frequency SNPs being more evident in the energy production‐related genes (Figure 2B).

If there is differential postlarval mortality among seep and control environments, or juveniles move preferentially to the environment to which they are better fitted, then the adults we analyzed do not necessarily represent the recruited larval population at the CO_2_ seeps, but the best‐fitted ones. In this case, the whole fSFS (control + seeps) would also present an excess of SNPs with intermediate frequencies, but the frequency of the alternative alleles would be different between control and seeps. We evaluated this possibility by testing differences in the allele frequencies of the SNPs detected in the candidate genes among seeps and control sites. The results indicated that there are no significant differences in the allele frequencies of the SNPs among the sites after correcting for multiple testing (*p*‐value > 0.05), suggesting no genetic differentiation between control and seeps adult fish for the up‐regulated genes in a reduced‐pH environment.

### 3.4 Fitness effects of the potential adaptive mutations to ocean acidification

Nearly half of the evaluated candidate genes (47%) presented one or more SNPs with intermediate frequency (MAF ≥ 0.30) based on the two methods of SNP detection. Six of these genes (*aoc2*, *hsp90ab1*, *rhag*, *agmat*, *rasa3*, and *taldo1*) have three or more intermediate‐frequency SNPs and were evaluated in more detail. The classification of these intermediate‐frequency SNPs in synonymous (i.e., not changing the protein) and nonsynonymous (i.e., producing amino acid changes), according to the related orthologous proteins in other species, showed that all but one are synonymous. This result suggests that the target mutations of adaptive selection to life at the reduced‐pH environment are not located within the coding sequences, but rather in neighboring linked functional regions such as regulatory sequences of gene expression.

Of the six genes presenting an excess of intermediate‐frequency SNPs, the *agmat* (*agmatinase*) gene was the only one with a nonsynonymous SNP in intermediate frequency. This SNP changes the ancestral glutamine for an arginine in the second position of the 135th codon of the orthologous *agmat* gene from *L*. *crocea*, and it is surrounded by other synonymous intermediate‐frequency SNPs (Figure S5). *agmat* produces an enzyme that catalyzes the agmatine, which has regulatory functions on different molecular targets including ion channels. Thus, mutations in the *agmat* protein could have important consequences on its activity and the downstream processes it regulates. Similar to the *agmat* gene, four other genes (*rhag*, *rasa3*, *aoc2*, and *hsp90ab1*) with an excess of intermediate‐frequency SNPs are also functionally related to homeostasis maintenance (Table 1). *rasa3* is also related to signal transduction function, while *hsp90ab1* is a molecular chaperone with many different functions such as response to estrogen and regulation of specific target proteins involved in cell cycle control and signal transduction (FK: homeostasis, energy production and protein binding; Table 1). Finally, *taldo1* is the one with similar elevated expression levels in both males and females from CO_2_ seeps and it is important in the metabolism of carbohydrates (FK: energy production; Table 1). Hence, these six genes with greatest evidence of an adaptive process to the reduced‐pH environment also show strong evidence of elevated expression at CO_2_ seeps (Figure S3D,F‐J) and are functionally related to energy production, homeostasis maintenance, and regulation of downstream biological processes.

## 4 DISCUSSION

Volcanic CO_2_ vents can act as natural laboratories to study molecular responses to reduced environmental pH in marine species, providing insights into their scope for adaptation to future ocean acidification. Fish from natural CO_2_ seeps showed increased gene expression levels across the gonad transcriptomes, suggesting an effect of ocean acidification on the overall regulation of gene expression. The elevated gene expression was more pronounced in males than in females from CO_2_ seeps, which could have reproductive consequences and is possibly linked to the higher reproductive investment in males (Nagelkerken et al., 2021) as Common triplefin males provide parental care of the nests (Wellenreuther & Clements, 2007). Among the genes that presented significant elevated expression in individuals from CO_2_ seeps compared with those from control areas, we found seven genes that are regulators of gene expression. We also detected elevated expression in genes with regulatory functions on downstream biological processes, such as *hsp90ab1*, *rasa3*, and *agmat*. Hence, the increased expression of these genes with regulatory functions can potentially trigger the overall elevated rates of gene expression observed in the fishes from CO_2_ seeps.

Nearly half of the genes with significantly higher expression in individuals (males and females) from CO_2_ seeps are functionally related to acid–base regulation to maintain pH homeostasis. This observation adds to the emerging view that some genes are being up‐regulated in response to the greater energetic demand of living in acidified conditions (Kelly & Hofmann, 2013; Strader et al., 2020). Indeed, maintaining pH homeostasis places greater energetic demands on individuals via increased production of proteins transporting ions within and among cells. Accordingly, we find elevated expression in genes involved in protein and energy production, suggesting an overall increase in metabolic rates in individuals living at CO_2_ seeps. Increased metabolism could potentially create energetic trade‐offs, where increased energetic investment in maintaining homeostasis occurs to the detriment of other important energy‐demanding physiological traits such as reproduction (Kelly & Hofmann, 2013). However, Common triplefins from these CO_2_ seeps experience higher food availability due to bottom‐up CO_2_ enrichment (Doubleday et al., 2019), and with their behavioral dominance over other fish species, they gain better access to food resources (Nagelkerken et al., 2017). Thus, individuals living at CO_2_ seeps can maintain physiological homeostasis and compensate for the increasing energetic demand by increasing their food intake or reducing their activity levels (Nagelkerken et al., 2021). This energetic compensation might not involve long‐term adaptation directly but involve mechanisms of physiological acclimatization and behavioral adjustments. Nevertheless, the increase in population size of Common triplefins within CO_2_ seeps (Nagelkerken et al., 2016) combined with the significant increase in the overall rates of gene expression suggests an adaptive response to the acidified environment through the regulation of gene expression.

We investigated the possibility of long‐term adaptation by searching for footprints of natural selection in the patterns of nucleotide polymorphism in the up‐regulated genes under reduced pH, as these genes are potential candidates to adaptive selection. We found a significant excess of intermediate‐frequency SNPs in these candidate genes when compared to neutral genes. Because neutral genes describe the expectations of genes without evidence of selective pressures for the reduced‐pH environment, this result is a clear indicator of an adaptive process for life in an acidified environment. The excess of intermediate‐frequency SNPs in the candidate genes is compatible with the expectations of balancing selection in its form of spatial heterogeneous selection (Hedrick, 2006, 2007; Levene, 1953). Because larvae recruit randomly among acidified and nonacidified environments, the adaptive alleles for reduced pH did not reach fixation (i.e., local adaptation) in the Common triplefins at White Island but rather are maintained as standing genetic variation within the whole population. However, these adaptive alleles provide advantages when fish are recruited and live in the acidified environment and therefore offer adaptive potential to future ocean acidification.

Most of the potential adaptive SNPs to a reduced‐pH environment that we found in Common triplefins do not change proteins but are likely to be mutations located in noncoding nearby genomic regions. Such regions contain transcription's promoters and enhancers (cis‐regulatory sequences) that in turn are responsible for the up‐regulation of these genes. Because mutations in DNA sequences are physically linked within chromosomic regions, natural selection favoring genetic variants in cis‐regulatory sequences at the acidified environment would increase the frequency of neutral linked SNPs in coding regions (i.e., synonymous mutations not changing the protein sequence) leaving a pattern of an excess of intermediate‐frequency SNPs such as that we observed. The fitness effects of mutations in regulatory sequences that enhance the transcription of genes responding to the reduced pH are more likely to be smaller than those of mutations changing proteins. Reduced pH could trigger the expression of just a handful of genes with regulatory effects on the expression of many target genes, creating a cascade of increased transcription through mutated regulatory sequences (Yosef & Regev, 2011), which would explain the observed increased transcription levels in fish from the CO_2_ seeps. These mutated regulatory sequences would not impact the fitness of the individuals carrying them when living in an ambient pH environment, but these might allow fine‐tuned physiological regulation in a reduced‐pH environment. This idea of adaptation through changes in regulatory sequences of gene transcription is supported by the growing evidence indicating that such changes are highly important in species evolution (Carroll, 2008; Holloway et al., 2007) and adaptation to local conditions (Fraser, 2013; Mack et al., 2018). Moreover, we find the footprint of natural selection on the pattern of nucleotide polymorphism in genes grouped by functional categories. This result agrees with the emerging view that adaptation is more likely happening through the adaptive selection of mutations with small fitness effects located in many functionally related genes (Pritchard & Rienzo, 2010). Thus, our results suggest that Common triplefins have adapted to life in an acidified environment through genetic changes in the cis‐regulatory sequences of several genes functionally important to respond to a reduced‐pH environment.

**FIGURE 3:**
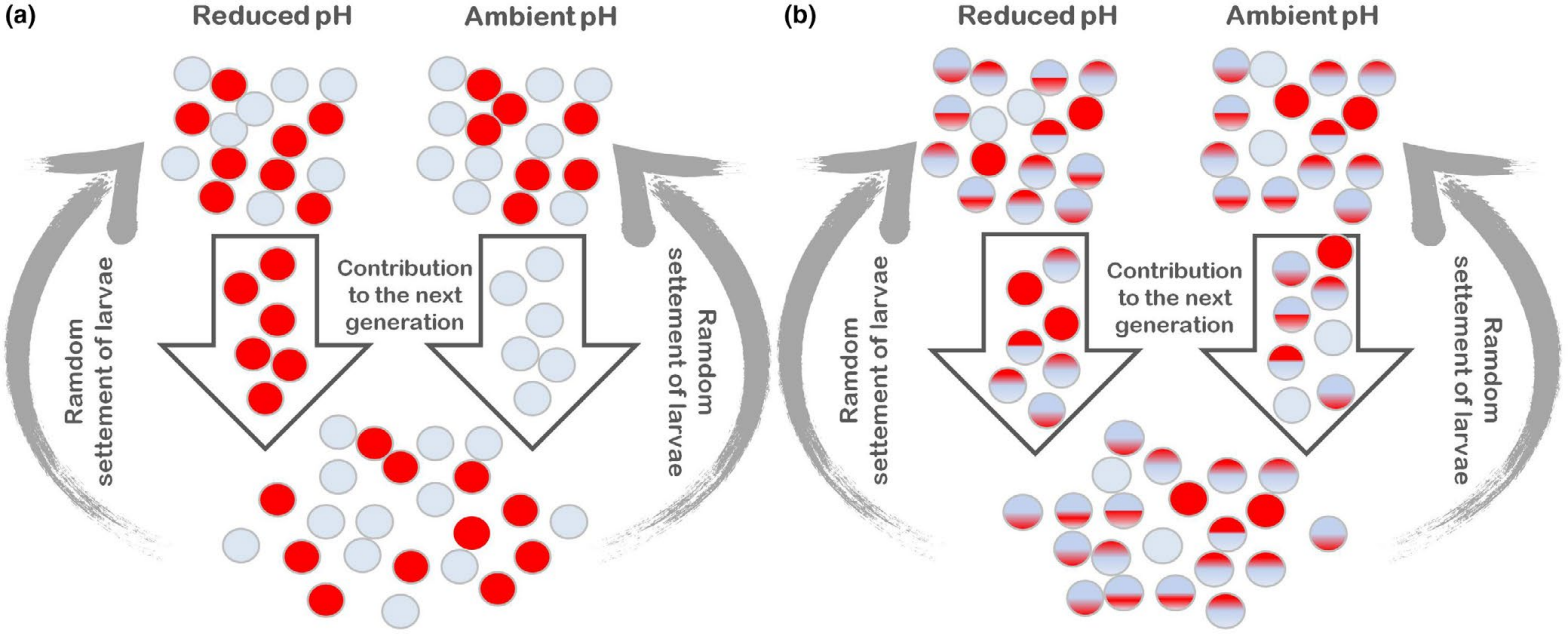
Models of potential adaptation to ocean acidification through balancing selection on (a) mutations with large fitness effects (e.g., affecting larval survival; modified from Palumbi et al., 2019) where individuals carry an allele of one mutation (phenotype represented by red circles) that is beneficial under reduced pH (i.e., increasing survival) but detrimental under ambient pH, (i.e., decreasing survival), and (b) polygenic mutations with small fitness effects (e.g., enhancers that increase gene expression in the reduced‐pH environment), where individuals carry some or several mutations that are beneficial under reduced pH. The amount of red color in the circles represents the different phenotypes produced by the different combinations of beneficial mutations. The genetic load or evolutionary cost in (a) will be higher than in (b), as many larvae that settle randomly in the different environments will not leave descendants (i.e., all gray circles in reduced pH and all red circles in ambient pH), while in (b), only a small fraction of the individuals will not be well fitted in either of the environments

The mechanism of adaptation we observed for the Common triplefin suggests some differences with current evidence of adaptation to acidified environments in other marine species. Evidence of balancing selection that maintains adaptive genetic variation to ocean acidification has also been found in wild populations of purple sea urchins (Brennan et al., 2019) and Mediterranean mussels (Bitter et al., 2019). These studies showed changes in allele frequencies due to selection on standing genetic variation that impacts larval survival after a treatment of reduced pH (Bitter et al., 2019; Brennan et al., 2019; Pespeni et al., 2013). As a consequence of this kind of selection, adaptation would be fast, but the adult population size is expected to decrease in the acidified environment. This decline in population size could have long‐term consequences such as loss of adaptive potential to other environmental conditions (Pespeni et al., 2013). However, in our case‐study, the Common triplefin shows an increased adult population size at CO_2_ seeps (Nagelkerken et al., 2017). Moreover, our results do not show evidence of differences in allele frequencies among seeps and control in candidate genes to adaptive selection, which suggests that these do not affect larval survival but most likely the physiological performance of adults under reduced pH. Hence, we propose a new conceptual model explaining how balancing selection could maintain the adaptive genetic variation to ocean acidification in Common triplefins, which is an alternative to that previously proposed for the purple sea urchin (Pespeni et al., 2013, Palumbi et al., 2019; Figure 3A). The new model involves adaptive selection of mutations with small fitness effects located in the cis‐regulatory sequences of several genes functionally important to respond to a reduced‐pH environment (Figure 3B). Different from the model proposed for purple sea urchin, this new model would release the Common triplefin population from the high evolutionary cost of maintaining beneficial mutations to reduced pH with large fitness effects (i.e., on larval survival). Thus, most of the Common triplefins from White Island are probably carriers of some or many beneficial genetic variants that might provide adaptation to ocean acidification through the regulation of gene expression.

In summary, our results indicate that adaptive genetic variation to reduced pH could be located in regulatory sequences of the transcription of genes responding to this environmental change. Regulatory mutations could potentially change the regulatory circuits of gene expression allowing fine‐tuned physiological responses to ocean acidification. Our case‐study in the Common triplefin shows that these adaptive mutations are maintained within the whole population at a local scale. Evidence of population structuring at regional scales has been found for Common triplefins (Rabone et al., 2015), and the degree of maintenance of adaptive mutations to ocean acidification at this scale deserves further studies in this species. However, the model presented here is applicable to all fish species with pelagic larvae and genetic flow among different environments. Acidification of the ocean is intensifying, and marine species tend to occur across wide geographical ranges with variable pH. Thus, it is likely that adaptive mutations allowing for life in slightly reduced‐ or variable‐pH environments already exist within local populations (Kelly & Hofmann, 2013; Palumbi et al., 2019; Strader et al., 2020). Highly dispersive marine larvae contribute to the flow of this local adaptive genetic variation among species populations, while balancing selection likely allows for the long‐term maintenance of this adaptive potential within populations. Hence, it might be expected that mutations in regulatory sequences of gene expression efficiently adjusting the physiological responses to reduced pH will become targets of pervasive adaptive selection in the near future under increasing ocean acidification.

## CONFLICT OF INTEREST

The authors declare no conflicts of interest.

## Supporting information

Figures S1–S5

Tables S1–S10

## Data Availability

The data that support the findings of this study are openly available at NCBI BioProject at https://www.ncbi.nlm.nih.gov/bioproject, with reference number: PRJNA658206.
