# Supplementary material for: Natural CO2 seeps reveal adaptive potential to ocean acidification in fish
Source: Evol Appl. 2021 May 5;14(7):1794–806. doi: 10.1111/eva.13239 (PMC8288007; doi:10.1111/eva.13239)
Supplement: Supplementary file 1 — Figures S1–S5 [file EVA-14-1794-s001.pdf]

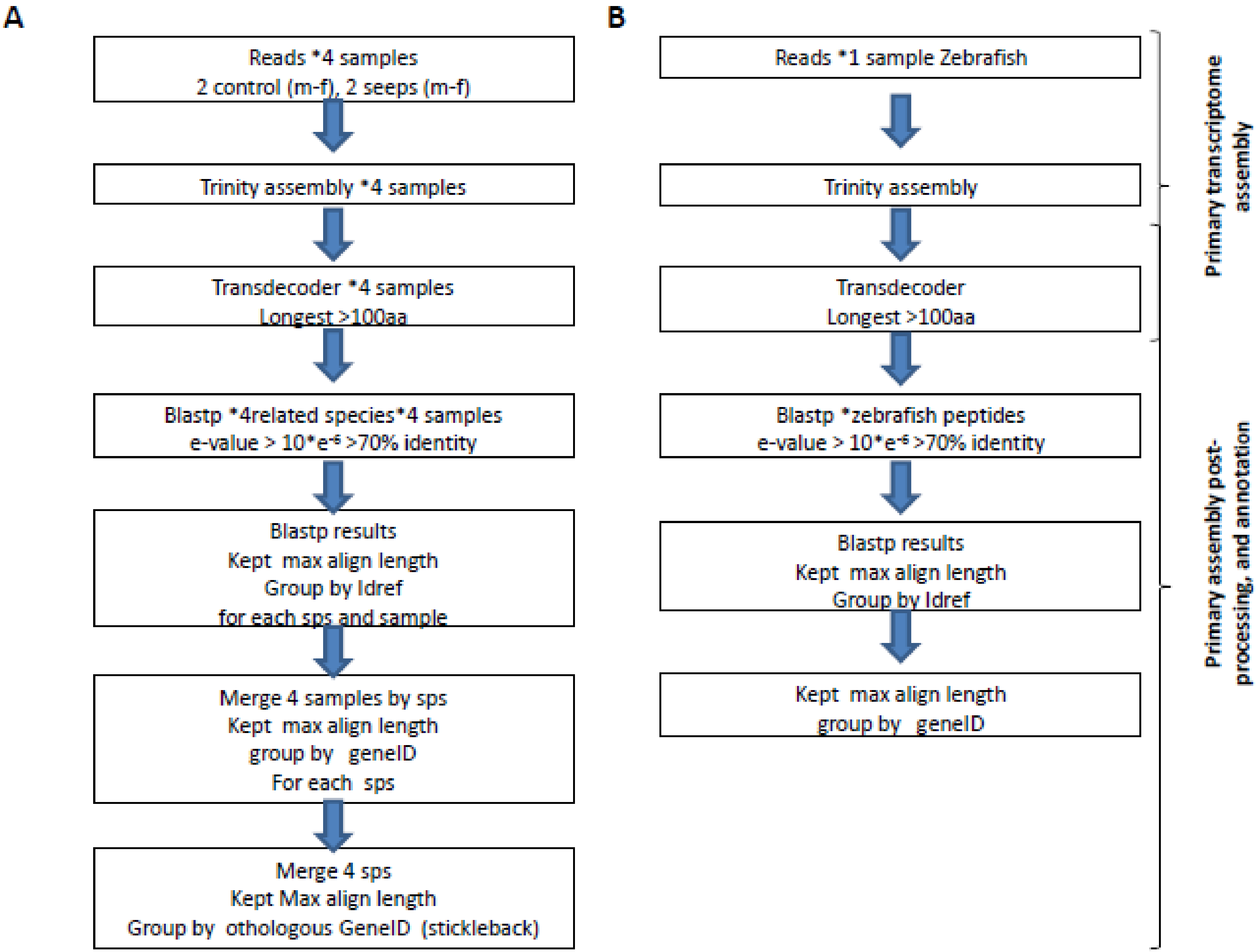

Suppl. Figure 1: Pipeline for de-novo assembly and annotation of common triplefin gonads transcriptome**: A)** common triplefin samples, **B)** zebrafish sample taken as control.

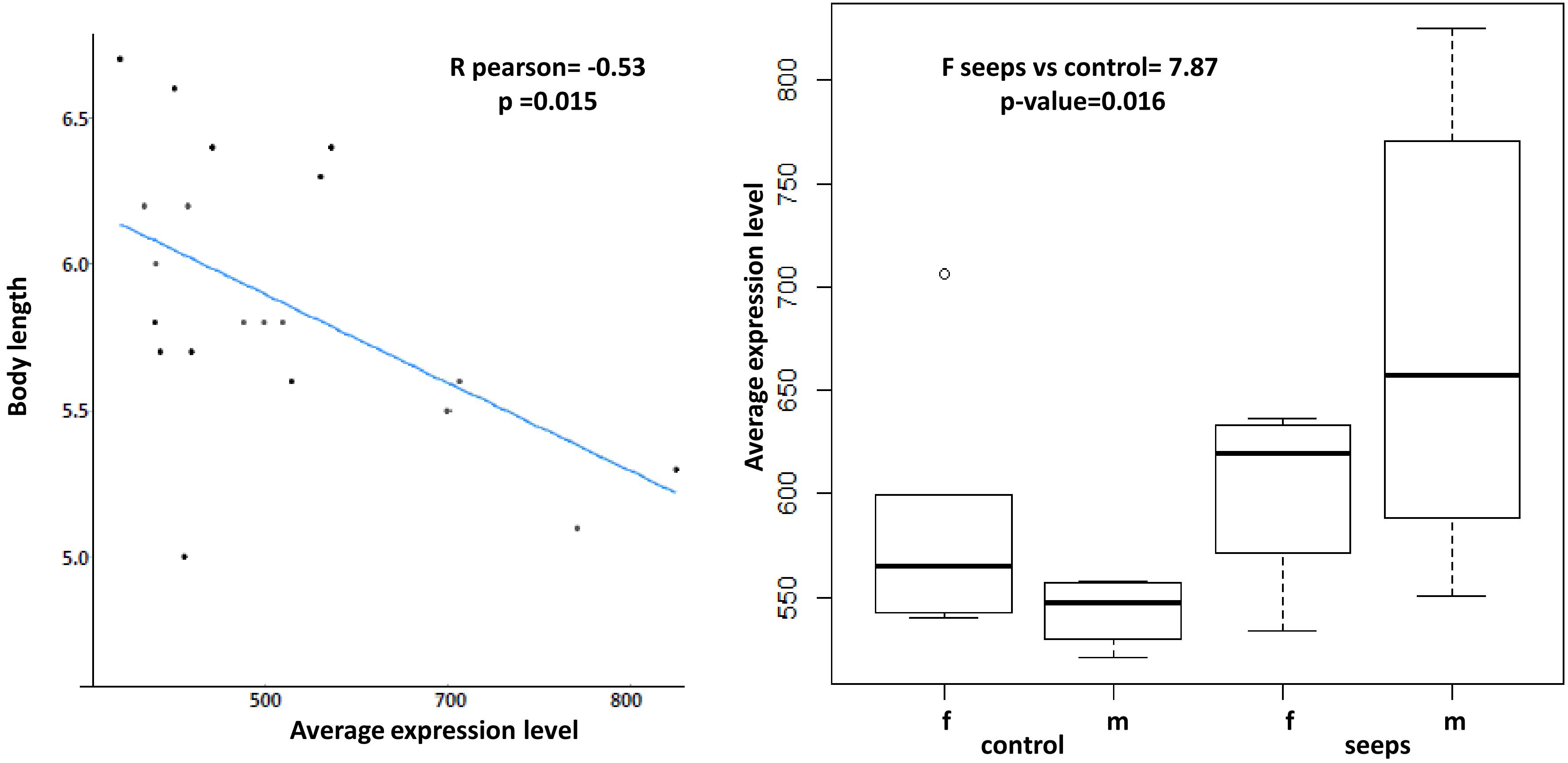

Suppl. Figure 2. A) Correlation between body length and mean levels of expression; **B)** Boxplot showing differences in levels of average gene expression among control and seeps fish (line:median,lower and upper hinges correspond to the 25th and 75th percentiles and whiskers are 1.5*IQR, dots represent outliers). f:female, m:male. Expression levels are expressed in numbers of normalized reads counts.

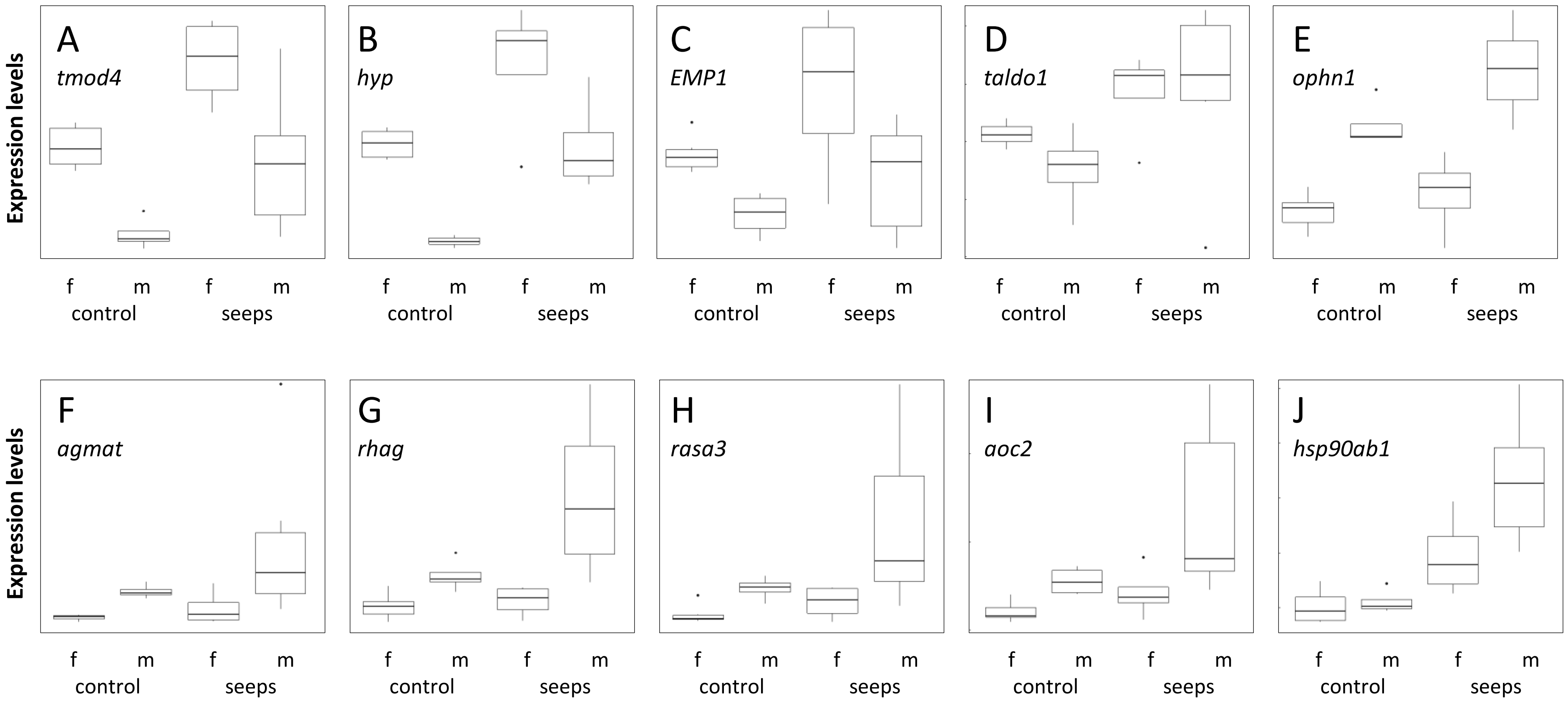

Suppl. Figure 3. Boxplots showing differences in gene expression of different up-regulated genes (line: median, lower and upper hinges correspond to the 25th and 75th percentiles, whiskers are 1.5*IQR, and dot represents outliers). f:female, m:male. Expression levels are calculated in numbers of normalized reads counts. A), B) and C) genes with higher expression in females than males from seeps, D) highly expressed gene with similar levels in both males and females from seeps. C) and E) genes with evidence of X-linked diseases in humans. D) and F) to J) genes with detected excess of intermediate-frequency SNPS. Note that Y axis has different scale for every gene (not presented). p-values and log2 fold changes obtained from the Likelihood Ratio Test (LRT) showing significant up-regulation in CO_2 seeps for each gene are presented in **Supplementary Table 9**.

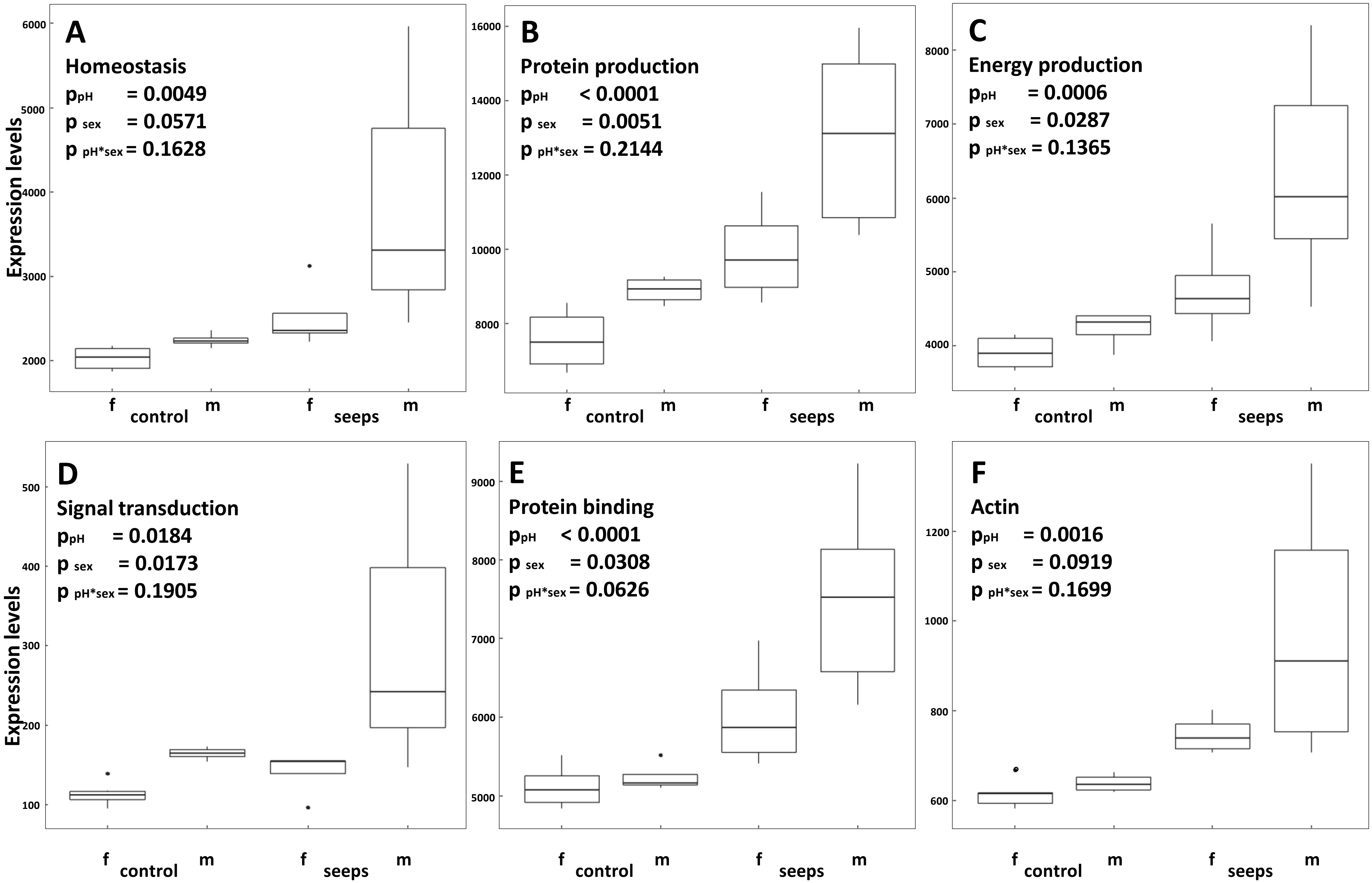

Suppl. Figure 4. Boxplots showing differences in gene expression of up-regulated genes grouped by functions (line: median, lower and upper hinges correspond to the 25th and 75th percentiles, whiskers are 1.5 * IQR, and dots represent outliers). f:female, m:male. Expression levels are expressed in numbers of normalized reads counts. No significant interactions were found between the factors environmental pH and sex for any of the cases. Note that Y axis has different scale for every group of genes.

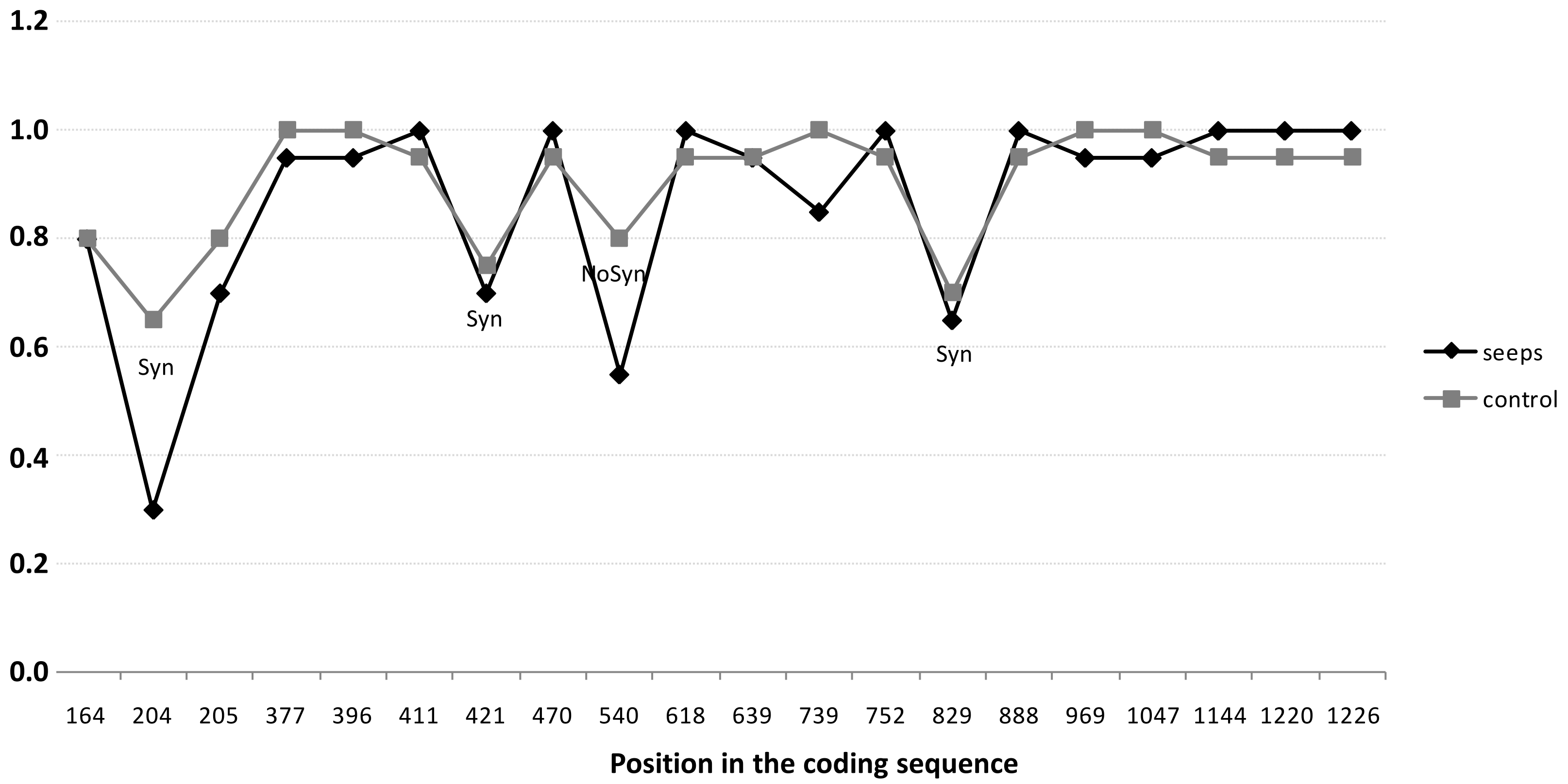

Suppl. Figure 5. Reference allele frequency of the SNPs distribution along the coding sequences of the *agmat* gene, showing the non-synonymous (NoSyn) and synonymous (Syn) SNPs with intermediate frequencies.
